# Using the cultural model to plan intervention for malaria control in immigrants and native communities in endemic area, earmarked for malaria elimination southeastern Iran

**DOI:** 10.1186/1475-2875-11-S1-P132

**Published:** 2012-10-15

**Authors:** Khandan Shahandeh, HamidReza Basseri, Esmaeil Shojaeizadeh

**Affiliations:** 1 Department of Health Education and Promotion, School of Public Health, Tehran University of Medical Sciences, Tehran, Iran; 2 Center for Community Based Participatory Research. Tehran University of Medical Sciences, Tehran, Iran; 3 Department of Medical Entomology & Vector Control, School of Public Health, Tehran University of Medical Sciences, Tehran, Iran; 4 School of Public Health, Tehran University of Medical Sciences, Tehran, Iran

## Background

To improve malaria control measures, taking into account local beliefs and practices are essential. Recently, Iran has been earmarked for malaria elimination while the majority of malaria patients are imported cases from eastern neighbouring countries.

In the present study, we employed the culture model as a theoretical framework to examine how health beliefs, behaviors and practices associated with improving access to prevention measures, early diagnosis and treatment of malaria in two communities, immigrants and native residents in a malaria endemic region located in southeast of Iran.

## Materials and methods

A mixed-methodology was designed by means of two quantitative surveys and qualitative focus groups. In total, 380 participants volunteered to take the cross-sectional survey, with 185 immigrants, 195 native residents completing quantitative surveys and also 40 participating in the qualitative focus groups.

## Results

A significant association between education level and knowledge on malaria transmission was also observed within both communities. Although the majority respondents associated the disease transmission with mosquito bites only 16.5% immigrants as compared to 63.4% native residents reported to use mosquito net. Data from focus group emerged three themes includes similarity in perception about malaria, difference in type of treatment and decision making and, finally resemblance to prevention of malaria in both communities.

## Conclusions

Matching the cultural characteristics of immigrants and native residents' culture to malaria interventions and services will improve receptivity to, acceptance of, and salience of these efforts.

## References

[B1] Onwujekwe O Uzochukwu B Dike N Okoli C Eze S Chukwuogo O Are there geographic and socio-economic differences in incidence, burden and prevention of malaria? A study in southeast Nigeria Int J Equity Health 2009 8 45 10.1186/1475-9276-8-45 20030827 PMC2806339

[B2] Airhihenbuwa CO Of Culture and Multiverse: Renouncing the ‘Universal Truth’ in Health Journal of Health Education 1999 30 267 273

[B3] Airhihenbuwa CO Healing our differences: The crisis of global health and the politics of identity 2007 New York: Rowman and Littlefield

[B4] Basseri H Raeisi A Ranjbar Khakha M Pakarai A Abdolghafar H Seasonal abundance and host-feeding patterns of anopheline vectors in malaria endemic area of iran J Parasitol Res 2010 2010 671291 21559055 10.1155/2010/671291 PMC2943101

[B5] Basseri HR Raeisi A Holakouie K Shahandeh K Malaria prevention among Afghani refugees in a malarious area, southeastern Iran Bull Soc Pathol Exot 2010 103 5 340 345 10.1007/s13149-010-0050-3 20526828

[B6] Martens P Hall L Malaria on the move: human population movement and malaria transmission Emerg Infect Dis 2000 6 2 103 109 10.3201/eid0602.000202 10756143 PMC2640853

[B7] Tatem AJ Smith DL International population movements and regional Plasmodium falciparum malaria elimination strategies Proc Natl Acad Sci U S A 2010 107 27 12222 12227 10.1073/pnas.1002971107 20566870 PMC2901446

